# Organoids in Translational Oncology

**DOI:** 10.3390/jcm9092774

**Published:** 2020-08-27

**Authors:** Marco Tatullo, Benedetta Marrelli, Caterina Benincasa, Elisabetta Aiello, Irina Makeeva, Barbara Zavan, Andrea Ballini, Danila De Vito, Gianrico Spagnuolo

**Affiliations:** 1 Department of Basic Medical Sciences, Neurosciences and Sense Organs, University of Bari “Aldo Moro”, 70124 Bari, Italy; danila.devito@uniba.it; 2 Marrelli Health—Tecnologica Research Institute, Biomedical Section, Street E. Fermi, 88900 Crotone, Italy; benedetta.marrelli@calabrodental.it (B.M.); caterina.benincasa@leverdipraterie.com (C.B.); elisa.aiello@tecnologicasrl.com (E.A.); 3 Department of Therapeutic Dentistry, Sechenov University Russia, 119146 Moscow, Russia; irina.makeeva@libero.it (I.M.); gspagnuo@unina.it (G.S.); 4 Department of Medical Sciences, University of Ferrara, 44121 Ferrara, Italy; barbara.zavan@unife.it; 5 Department of Biosciences, Biotechnologies and Biopharmaceutics, University of Bari “Aldo Moro”, Campus Universitario “Ernesto Quagliariello”, 70125 Bari, Italy; andrea.ballini@uniba.it; 6 Department of Precision Medicine, University of Campania “Luigi Vanvitelli”, 80138 Naples, Italy; 7 Department of Neurosciences, Reproductive and Odontostomatological Sciences, University of Naples, 80131 Naples, Italy

**Keywords:** translational medicine, scaffolds, tissue engineering

## Abstract

Translational medicine aims to translate the most promising preclinical research into clinical practice. Oncology is a continuously growing medical field: the scientific research on cancer biology is currently based on in vitro experiments, carried out on tissue culture plates (TCPs) and other 2D samples. In this context, 3D printing has greatly improved the biofabrication of new biological matrices that mimic the extracellular environments, which may characterize healthy from cancerous tissues. Organoids have recently been described in several reports on scientific literature. The term that better describes such organoids-based tumoral tissues is “tumoroids”. Tumoroids are substantially “tumor-like organoids”, typically deriving from primary tumors harvested from patients. This topical review aims to give an update on organoids applied in translational medicine, paying specific attention to their use in the investigation of the main molecular mechanisms of cancer onset and growth, and on the most impacting strategies for effective targeted therapies.

## 1. Background

Recent developments in the fields of cell biology and tissue engineering have improved all the medical fields, creating interesting opportunities to study some key-processes that are deeply involved in several human pathologies. Organoids are self-organized cell aggregates derived from primary tissue or mesenchymal stem cells (MSCs) that are capable of self-renewal, typically organized in three-dimensional (3D) constructs able to replicate the complex structure of an organ, mimicking its in vivo physiology. Organoids are useful tools to investigate several aspects of tissues and organs in vitro [1,2]. Recently, human organoids have been used to build customized models of specific human diseases. Moreover, organoids can also give important information on stem cell behavior and fate [3,4]. In this landscape, several research groups are working on innovative organoids-based cancer tissues models, called “tumoroids” [5,6,7].

The term “tumoroid” means “tumor-like organoid”: tumoroids typically derive from primary tumors harvested from oncological patients [8].

The knowledge of cancer physiopathology can be improved through specific studies on some important aspects characterizing the tumor microenvironment (TME). Furthermore, the pathways involved in cancer onset and cell-to-cell communication, as well as the role of TME in cancer progression, are still not fully understood; thus, tumoroids may have a strategic role in cancer research.

Researchers have investigated tumoroids grown with different cancer cells and MSCs, and cultured on different scaffolds and resorbable matrices: the main aim was to reproduce reliable 3D models of several types of cancers [2].

Tumoroids are typically clustered in 3D structures, totally grown in vitro: these structures can organize themselves into 3D organoids similar to the primary tissue where cells have been harvested [9,10]. Tumoroids can mimic human TME; nowadays, they are considered a promising tool for cost-effective studies on novel anticancer drugs to be used in precision medicine [11].

Over the years, several promising techniques have been used to fabricate 3D scaffolds for cell culture. A well-known strategy was to put cells in “spinner flasks” under continuous stirring: this strategy has allowed the creation of spherical clusters of cells, growing drop-by-drop; however, this technique required huge quantities of culture medium and the availability of specific equipment. 

Alternative techniques, called 3D micro-molds, were also investigated to overcome the high culture medium consumption highlighted in the previous method: these techniques were able to produce clusters with different shapes, not only spheroids [12].

The first studies on long-term cultures of 3D spheroids demonstrated that they frequently resulted in a compact syncytium of cells unable to ensure a proper diffusion of nutrients and oxygen into the central zone of these 3D structures [2]. Conversely, the use of 3D supports in Good Manufacturing Practice (GMP) rooms has improved the viability of such tumor-based spheroids. Recently, extracellular matrices from tumor tissues have been also used to promote the releasing in situ of extracellular vesicles (EVs), micro-RNAs (miRNAs) and other molecules [13]. Of course, the use of bioreactors and 3D-bioprinting has provided customized bio-scaffolds, more complex than spheroids, able to interact with cancer cells and to actively modify their behavior [14,15]. 

## 2. Translational Use of Organoids 

In the last decades, several research teams have worked on embryonic stem cells (ESCs), adult stem cells (AdSCs) and induced-pluripotent stem cells (iPSC). Regenerative procedures have allowed researchers to differentiate stem cells towards almost all the endodermal (lung, stomach, liver, small intestine) and ectodermal (brain and retina) tissues in vitro [1]. Organoids, under specific culturing conditions, are able to self-renew and differentiate towards several phenotypes in vitro. Similarly, tumor-like organoids can reproduce the complexities observed in human cancer tissues [2]. 

Cancer organoids can also be fabricated starting from cryopreserved tissues, usually stored in liquid nitrogen for research reasons [6]. Organoids are grown as cells embedded within drops of medium, and then clustered to slowly grow into 3D structures. Organoids are usually cultured on extracellular matrix (ECM) niches: after the early growth and differentiation, they are then organized into small heterogeneous clusters of cells via enzymatic digestion or mechanical disruption; these clusters are able to self-develop organoids themselves, reproducing the primary tissues [5].

Three-dimensional culturing condition can be easily achieved within hydrogel-based matrices, containing a gelatinous mixture made of extracellular matrix compounds (including laminin, collagen, and proteoglycans) and growth factors (including TGF-β and FGF). Cells cultured to create organoids undergo cell-to-matrix and cell-to-cell interactions [7]. 

Synthetic hydrogels are soft gelatinous matrices made of synthetic polymers, such as polyethylene glycol (PEG), polylactic acid (PLA), or poly(vinyl acetate) (PVA). On the other hand, polymeric hard scaffolds are an important tool for the research on cell-to-ECM interactions [16].

**Figure 1:**
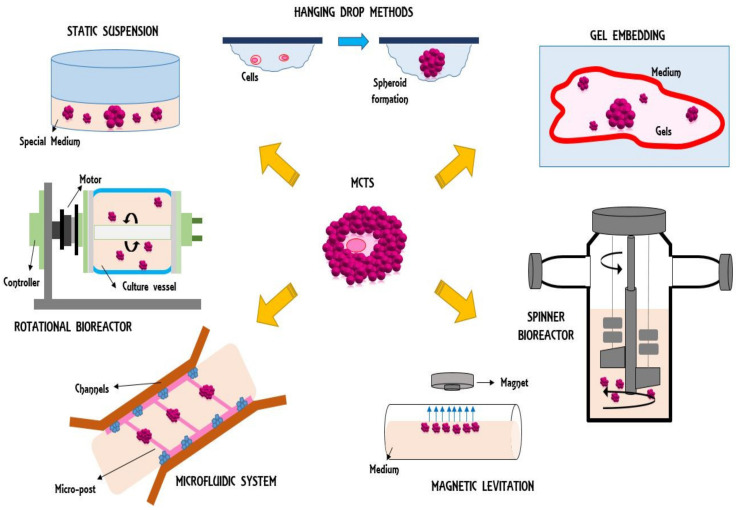
Synoptic view of 3D organoid fabrication methods.

Three-dimensional organoids are mainly grown via static suspension, hanging-drop method, magnetic levitation, spinner bioreactor, rotational bioreactor, microfluidic system, and gel embedding techniques (Figure 1) [14].

These methods recognize different basic concepts; moreover, the fabrication technique concretely influences the development of different microenvironments characterizing some developmental aspects of tumoroids. Up to now, organoids cultured in bioreactors seem to ensure higher cell viability; moreover, the ECM surrounding the cells is well organized: probably, the homogeneous flow of culture medium and nutrients around the cells is able to improve the overall growth. This process ensures a high-rate of medium replacement at the surface of the organoids, reaching the core of the organoid [11,16,17,18,19].

An interesting approach for organoid culturing involves hydrophobized surfaces for cell culture [5]. Physiologically, the surface tension creates hanging drops that develop spheroids [15,16].

Recently, a new technology has been described: the piezoelectric inkjet printing (IJP). It holds several advantages that can be used in tumor research. IJP works using piezo-crystals that create nanoscale droplets (drop-on-demand inkjet process); IJP can fabricate precise patterns of cells combined with hydrogel-based matrices at high speed (10 to 1000 Hz) and at high resolution (≤100 μm) [8]. 

The research on 3D organoids have carried out a number of results used to better understand several pathways in cell biology; interestingly, research on organoids demonstrated that CRISPR-associated protein-9 (clustered regularly interspaced short palindromic repeats(CRISPR)/Cas9) can modify human genome in vitro [20], and is able to modulate the off-target effects on the organoid genome [21]. Thus, organoids are a translational tool used to generate reliable preclinical models for several diseases. 

## 3. Comparison of Characteristics between 2D vs. 3D-cultured Tumor Cells

The scientific research on cancer biology is mainly based on in vitro experiments carried out on 2D tissue culture plates (TCPs) and on histological samples. However, 2D cultures often fail to provide important information on 3D aspects of cell-to-cell interactions. Studies have also demonstrated that 2D cell culture fails to accurately reproduce the organs physiology and is not able to give information on novel drugs [2]. Cells cultured on 2D supports are exposed to homogeneous environments, growth factors, nutrients, oxygen, and percentage of CO_2_ [22]. Conversely, cells within solid 3D tumors are exposed to different gradients of biochemical molecules and biological signals, which can promote both stimulatory and inhibitory effects on tumor progression [16]. 

For these reasons, results achieved with 2D-based research cannot be translated into in vivo settings [5]. In fact, cancer progression is a highly dynamic process including several factors, such as change in pH, cell-to-cell paracrine signals, and impaired gene expression; moreover, cancer cells are exposed to chemical and biological signals which can exert both stimulatory and inhibitory effects on tumor progression exposed to gradients of critical chemical and biological signals leading to both stimulatory and inhibitory effects on tumor progression [5,23]. Nevertheless, the lack of the third dimension can mask the experimental observations, generating misleading and contradictory results [5].

Tumoroids are 3D models developed to mimic the in vivo tumors [22]. In vitro 3D organoids hold several advantages, compared to traditional in vivo animal models or other traditional 2D cell culture techniques. First of all, 3D organoids can develop and expand in the third dimension, and thus they can ensure better tissue and organ development, with morphological features found only in in vivo models [17]. Importantly, the alteration of the 3D structure of extracellular matrix (ECM) plays a pivotal role in cancer physiopathology: in this light, cell culture on TCPs may affect the whole research on cancer metabolism and treatment [6]. The fabrication of complex biological matrices, mimicking the extracellular microenvironments that may characterize healthy from cancer tissue, has greatly been improved by 3D printing: the reproduction of an analogous tumor environment is strategic to designing successful therapeutic strategies. ECM can be described as a 3D network of macromolecules, mainly sustained by subsets of collagens, proteoglycans/glycosaminoglycans, elastin, fibronectin, laminin, and other glycoproteins, with different physical, mechanical, and biochemical functions [24]. Moreover, ECM works as a ligand able to regulate cellular function, growth, and differentiation, also acting as a reservoir for bioactive molecules. Finally, ECM is fully involved in tissue architecture, and can be useful in the building-up of tissue-specific scaffolds [24]. 

Gel-free tumoroid models cultured on NanoCulture Plates (NCP) and ultra-low attachment (ULA) plates seem to be highly versatile, having undoubted advantages in secretome collectability and in vesicles releasing [22]. A recent study on metastatic cancer cells using 3D organoids developed in stemness-enhancing medium, compared with standard 2D cultures, showed that 3D tumoroid models increased the tumorigenesis in vitro, contributing to a deeper understanding in tumor progression [25].

In this landscape, 3D organoids derived from a human organ can stably maintain their characteristics, even after several manipulations, without any significant genetic or physiological changes [18]. Moreover, some organoids, such as the gastrointestinal (GI) organoids, can easily be established by isolating epithelial crypts from a routine GI biopsy, and GI organoids can grow and differentiate into crypt-villus structures within only 10 days. Finally, the ease of managing such 3D structures is demonstrated by the ability showed by intestinal stem cells (ISC), such as *Lgr5+* or *Bmi1+* stem cell, which can generate continuously expanding, self-organizing organoids also without any subepithelial cellular niche [19]. 

## 4. Tumoroids in Cancer Research

A deep knowledge of the local microenvironment in cancer onset may lead to controlling the fate of cancer cells. Breast cancer, for example, develops into a highly structured ECM, creating complex interactions among cells and ECM through direct contact and signaling molecules [18]. The breakdown of the ECM may create the proper conditions to stimulate specific factors supporting the onset of cancer [11]. To better investigate this aspect, tumoroids have been created using breast cancer cells and ECM: these compounds were able to induce a massive differentiation and growth of stem cells obtained from other organs after their injection into the mammary glands of mice [14]. Tumoroids have been used to understand if the healthy mammary microenvironment was able to induce a physiological behavior in breast cancer cells, and the response of cancer cells to new drugs [26].

In another study, tumoroids deriving from primary and metastatic colorectal adenocarcinoma were used to study how K-RAS mutation (Kirsten rat sarcoma mutation) affects colorectal adenocarcinoma growth. The K-RAS protein contributes to the transmission of growth signals in the nucleus of cells, leading to an increased cell growth [27]. The K-RAS pathway is amplified in colorectal cancer: in fact, abnormal K-RAS mutations can induce the hyperproliferation of the epithelium, resulting in the development of infiltrating adenocarcinomas [28]. 

In vitro investigations into tumoroids have clearly demonstrated that cancer cells cultured on 3D scaffolds derived from autologous ECM are fully able to reproduce the same microenvironment developed in vivo. Of course, the availability of reliable and complex cancer models working in safe and controlled experimental conditions has created the right conditions to test the effectiveness of novel chemotherapies, anticipating their side effects, and the activation of harmful immune reactions [27]. (Table 1)

Recently, the analysis of tumoroids from patients affected by colorectal cancer (CRC) showed a common molecular pattern in all the samples investigated; moreover, the authors reported that tumoroids were also responding to chemotherapy in the same way observed in the cancers treated in vivo. Small samples of human-derived tumoroids have been injected into murine models: after a brief engraftment time, tumoroids developed invasive and aggressive colorectal cancers, with metastases in the lungs and liver. These tumoroids engrafted on murine colon mucosa were treated with novel drugs, showing therapeutic effects completely comparable with those reported on humans [29] (Table 1).

Scientists have used tumoroids-based models to perform in-depth investigation into such pathways involving the immune system cells in cancer progression: this strategy allowed assessment of the impact of different chemotherapies on the immune reply, to calibrate the dose-effect percentage on aggressive cancers [30].

Tumoroids have also gained an important role in the study of brain tumors. An in vitro 3D study-model called neoplastic brain organoid (NeoCOR) was performed on this topic: the invasiveness of tumor and the effects of drugs were investigated on cells treated with CRISPR-Cas9 to carry specific mutations. The NeoCOR model achieved the reproduction all the tumor environments related to brain cancers [31].

Researchers have successfully generated “mini-tumoroids” (up to 0.5 mm), to study liver cancers. Thus far, 29 new drugs were tested on liver-cancer mini-tumoroids: a new drug was found to inhibit the activation of the ERK protein, gaining interest for future liver cancer therapies [32]. Following this in vitro study, mini-tumoroids were injected into mice livers, then treated with the newly discovered drug: the in vivo results showed a significant reduction in tumor growth in those mice treated with the new drug, thus confirming the reliability of the in vitro preliminary tests achieved with tumoroids [32]. (Table 1) This reliability has been further confirmed in other studies, such as a clinical trial conducted on oncological patients treated with radiation therapy and with chemotherapy for colorectal cancer: here tumoroids were able to reproduce in vitro the same biological effects reported on cells/tissues of patients [33].

Tumoroids have been strategic to disclosing the interactions among cancer cells, autologous mesenchymal stem cells (MSCs,) and local ECM [34]. Nevertheless, a recent study on pancreatic cancer, has also investigated induced pluripotent stem cells (iPSCs): briefly, the combination of stem cells and iPSCs was able to perfectly reproduce functional pancreatic cells within their stroma, and these cells were used to study their interactions with cancer cells [35]. 

Stem cells isolated from cancer tissues can themselves differentiate into tumor-like tissues; however, the behavior of such cells seems to be influenced by the local environment. In a study conducted in 2018, researchers investigated the behavior of stem cells from cancer tissues grown in two different experimental conditions: on standard tissue culture plates (TCPs) and NanoCulture Plates (NCPs); stem cells grown on NCPs created tumoroids with tighter intercellular adhesions, and they also showed morphologies and superficial markers exactly reproducing the characteristics of primary tumors. On the contrary, stem cells grown on TCPs expressed genes associated with cell differentiation. The authors also found that stem cells grown on NCPs were able to release exosomes containing epithelial cell adhesion molecules (EpCAM) and heat shock protein 90 (HSP90) that have been closely related to an increased growth of cancers [36].

## 5. Features of 3D Tumoroids

The organoid is an in vitro 3D cell cluster deriving from stem cells or progenitor organs that behave similarly to the counterpart in vivo both in morphology and in functionality [37]. The main feature of 3D tumoroids is the ability to self-organize and segregate cells to form structures with histogenic properties similar to those in vivo, which is called self-organization [38].

The cultivation of tumoroids in clinical research allows the possibility of cultivating several different cell types belonging to the same organ together. The possibility of directly cultivating different cells allows the researcher to investigate the characteristics of an in vivo system, thus referring to all the cells that compose it and being able to analyze their interactions accordingly. [37].

Physiologically the neoplastic cells within a tissue are organized in a complex three-dimensional network, stabilized by nutrient gradients and signal transduction mechanisms determined by cell–cell and cell–extracellular matrix–cell contact. The models generated thanks to the three-dimensional culture systems can satisfy the aforementioned requirements and can therefore be used as preclinical tumor models [39]. Multicellular spheroids can contain an extensive extracellular matrix that determines a network of connections not only cell–cell but also cell–matrix responsible for the penetration and action of drugs. The particular in vitro interaction of the 3D model also affects the distribution and function of biological effectors, such as hormones and growth factors, which regulate the mechanisms of growth, differentiation, and cellular death. These models more accurately reflect the characteristics in vivo not only at a biochemical and mechanical level, but also at the level of gene and protein expression [40].

Three-dimensional systems engineered through nanoimprinted scaffolds, according to Yukie Yoshii et al., in a work published in early 2011, have better reproduced the native tumor microenvironment favoring growth, migration, and intercellular adhesion. The formation of the spheroids starting from the sowing of the MSCs on the scaffolds has also preserved and optimized cell proliferation and viability. The same authors of the experimental study also concluded by stressing how these models are useful for delineating the biological mechanisms that regulate the pathological anomalies observed in cancer. Nanoimprinted scaffolds are used as 3D culture models to facilitate spontaneous tumor cell migration and well-regulated spheroid formation. [41]. Furthermore, these 3D tumoroids are characterized by the absence of innervation and vascularization, their three-sheet structure allows for long cellular maintenance, high genetic stability, and possible cryopreservation [42].

The latest studies on tumoroids confirm their use in research on the toxicity of nanomaterials. Through the use of 3D structures, the researchers would seem to have designed the nanoparticles for the specific targeting of tumors avoiding systemic toxicity, the degradation of the NPs within the body, and the accumulation in the internal organs without affecting the biological processes. In particular, ex vivo organoid models can be used in the early stages of the development of nanopharmaceuticals [43,44].

## 6. Applications of Tumoroids as a Study Model

Tumor microenvironment (TME) is a key factor in tumor growth and invasiveness. TME can have a different role in each different tumoral form; in this context, recent therapeutic models have been targeted at the TME to better understand the role of nanovesicles and local factors on cancer response to therapies [45]. An important issue is to understand if and how TME is able to impact genetic patterns of cancer cells, targeting future therapies towards the tissue where the tumor is located [46]. Usually, in solid tumors, the core is populated by cytotoxic lymphocytes; some forms of solid tumors have a scarce presence of inflammatory infiltrate: they are termed infiltrated-excluded (I-E) tumors. I-E environments have been associated with various epithelial cancers, such as colorectal carcinoma, melanoma, and pancreatic ductal adenocarcinoma [47]. I-E tumors have been hypothesized to be poorly immunogenic or “cold” [48]. Compared to other forms rich in inflammatory cells, I-E tumors seem to show a reduced activation of surface markers, resulting in an adaptive immunity unable to recognize and respond to any pathological condition [49]. On the other hand, infiltrated-inflamed (I-I) tumors have TMEs rich in inflammatory and immunological cells: they are called “hot” tumors and are characterized by high infiltration made prevalent by cytotoxic lymphocytes [50].

In this complex and heterogeneous crosstalk among cells, TMEs, and molecular mediators, we can recognize some specific and strategic pathways that represent a target for studies on cancer therapy. The mitogen-activated protein kinases (MAPK) pathway, also known as the RAS-RAF-MEK-ERK signal cascade, transmits upstream signals to its effectors to regulate several physiological processes, such as cell proliferation, differentiation, survival, and death. The MAPK pathway is the most frequently mutated signaling pathway in human cancers, thus it can be considered a promising target for cancer therapy [51,52]. Targeted therapy against RAS-RAF-MEK-ERK, tyrosine kinase inhibitors, and other small molecules, has been associated with an early therapeutic effect; unfortunately, this effect has shown a limited time of efficacy. Conversely, treatments targeted against immune checkpoints (anti-CTLA-4 and anti-PD1/PDL1) seem to work better if applied as long-term treatments [52,53].

Nowadays, the scientific literature strives to obtain robust clinical data to assess the best therapeutic pathways. In this context, an interesting approach has been focused on the use of anti-angiogenic agents working as a targeted immunosuppressors within the TME [46,48]. There is preclinical and clinical evidence suggesting that angiogenesis and immunosuppression are processes that share the same local biological regulators. In fact, the presence of angiogenic molecules, such as vascular endothelial growth factor (VEGF) and angiopoietin 2 (ANG2), within the TME may better drive immune cells towards the tumor, by altering the expression of adhesion molecules on endothelial cells (ECs) and immune cells [46].

Starting from these preliminary outcomes, there are several ongoing clinical trials aimed at investigating novel targeted therapies on tumoroids [9]. Strong data on tumoroids have been obtained in a multicenter observational cohort study: the TUMOROID RCT (NL49002.031.14), which has involved patients with metastatic colorectal, breast, and non-small-cell lung carcinoma (NSCLC) [9]. In this trial, clinical data have been collected and compared with the in vitro data obtained by tumoroids from the same patients. Early data report contrasting hypotheses; nevertheless, the final results are far from being fully analyzed and critically evaluated, and there are several issues about the differences of types and duration of tested therapies. Moreover, marked differences have been also pointed out on the toxicity of several treatments; emerging attention has been given to the immunomodulatory mechanisms observed in TMEs of tumoroids. In their study, Vlachogiannis and colleagues [12] developed organoids from 110 different patients (PDOs): patients were mainly affected by colorectal and gastroesophageal cancers. The phenotypic and genotypic profiles of PDOs were compared to the profiles of the primary tumors, showing an overall overlapping among them. PDOs were able to simulate the efficacy of different drugs on tumors collected: the success rate of drugs tested on tumoroids and patients was more than 88% in both cases. The superimposable outcomes of the studies on PDOs and humans are encouraging and confirm that PDOs may be an ideal platform to identify and test the best anticancer drugs. In conclusion, the utility of tumoroids as applied study models has been limited to preclinical evaluations on therapeutic approaches and on their ability to show interactions among cells, drugs, and TMEs in reliable cancer models [10]. One of the most appreciated characteristics related to the studies on tumoroids is their availability to be cultured directly from patient samples within a few days, rather than investing months for research on mice or other animal models.

## 7. Applications of Tumoroids for Drug Screening and Drug Discovery

Organoids/tumoroids, based on 3D culture systems, are a topic of general interest, as they are also useful for drug screening and discovery [54]. Screening tests must be highly reliable: currently, the most used is called HTS, or high-performance tests. HTS includes both biochemical and cellular tests, and it works by mimicking the tissue, expressing specific factors that activate specific signaling pathways. Three-dimensional cultures not only have these characteristics but can also reproduce cellular responses to drug treatments. Tumoroids have a high usefulness in drug testing and discovery, as they can reproduce the invasive behavior of human cancer cells, mimicking cell-to-cell and cell-to-ECM interactions, so creating the ideal microenvironment for in-depth investigations of all those mechanisms acting between tumor and stromal components [55].

Three-dimensional culture methods are numerous, and some types are still evolving; some novel methods can produce avascular tumors, and other methods on the contrary allow good investigation of tumor angiogenesis. Therefore, the type of drug to be discovered and the type of anti-cancer therapy to be tested need a strong cell model, and must be easy to use and reliable. Recently, two different breast cancer cell lines behaved differently, even if cultivated in the same way; in more detail, MCF7 cells created tight 3D spheroids on p-HEMA coated plates, while SKBR3 cells only created cell aggregates. Obviously, the setting up of 3D cultures is much more complex than 2D; nevertheless, the possibility of being used to investigate 3D cell aggregates in vitro allows researchers to shorten the time taken to obtain precious information on efficacy and toxicity of multiple drugs, obtaining accurate results that strongly direct the clinicians towards those specific drugs that show stronger effects on the cell targets [56]. Sogawa and colleagues examined six pharmacologically active compounds that potentially reduced the viability of 3D tumoroids; such compounds were AL8810, AP5, ART, Benz, chlorzoxazone, and carboplatin, and they were tested in order to understand their role in anticancer therapy. In this study, the technique used was a complex system of tumoroids combined with matrix metalloproteinase 9 (MMP9) that promoted a specific fluorescence useful to track both the morphology and progression of tumoroids. [57]. Complex tumoroid systems were also used to test the effects of a CDK2 inhibitor on the suppression of epithelial-mesenchymal transition (EMT) in aggressive cancers. The effectiveness of this new class of drugs in controlled trials was investigated [57].

MMP9 is one of the markers for aggressive tumors [58]; in his study, Sogawa reported that Benz inhibited the expression of MMP9, also reducing cancer metastases and angiogenesis.

The activity of MMP9 was also evaluated in another study by Sogawa. In this study, the authors developed a novel 3D tumoroid model, using mouse-derived and human-derived tumor cells together; the main results reported information about the pharmacological activity of dexamethasone and hydrocortisone on cancer cells, pointing out that steroid drugs were able to inhibit MMP9 activity without reducing local tumorigenesis. In this study 3D tumoroids from different tissues were combined, allowing measurement of the local tumorigenesis and invasiveness, also investigating the efficacy of drugs working on MMP9 gene amplification and β-catenin/MMP regulation [59].

Although a simple, fast, and cheap 3D model is still missing, three-dimensional models are preferable and reliable compared to 2D systems; in fact, in 2D systems, although the cells can grow rapidly, the same cells seem to have morphology different from cells from primary tissue [60].

## 8. Tumoroids in Oral Cancer Research

Oral cancer is one of the most impacting oncological diseases; it can develop on oral and maxillofacial tissues [53,61,62]. Oral squamous cell carcinoma (OSCC) represents 2% of all oral tumors; nevertheless, it has a much higher proliferation rate than the other tumors affecting the oral cavity [63].

The research is fully committed towards novel therapies against oral cancer; recently, tumoroids have also gained growing interest in this field. Hill et al. reported that tumoroids from oral cancer cells were able to express the molecular and genetic characteristics of the in vivo tumors, aiding researchers in the characterization of the efficacy of several novel drugs on oral cancers [64].

Driehuis et al. recently published a study on 31 different cell lines developing tumoroids of head and neck squamous cell carcinoma (HNSCC). Novel drugs have been tested on these tumoroids: interestingly, several tumoroids seemed to be selectively sensitive to such new drugs, thus suggesting a personalized approach to HNSCC therapy [65,66]. Driehius et al. also investigated whether photodynamic therapy (PDT) may influence the expression of epidermal growth factor (EGFR), which seems to be correlated with OSCC onset and progression. The abovementioned tumoroids were grown in parallel with healthy models: both the organoids and the tumoroids were exposed to PDT. The results showed that organoids from healthy tissue had lower EGFR expression levels, compared to those observed in OSCC tumoroids. Tumoroids were a useful disease model to also test alternative therapy based on PDT, as the authors claimed that PDT correlated with EGFR levels in OSCC patients [64,65,66,67].

Tumoroids have been demonstrated to reproduce structural and molecular environments surrounding the native tumor, thus allowing investigation of the most effective therapeutic strategies against specific pathways. Interleukin (IL-)6 is a pro-inflammatory cytokine within the *IL*-*6* family: IL-6 receptors have been correlated to esophageal and HNSCC tumors. Recent studies on HNSCC tumoroids have confirmed that tocilizumab, an anti-IL6Rα antibody, was able to significantly suppress tumorigenesis by inhibiting STAT3 and MEK/ERK signaling. Tumoroids were thus used to test new targeted therapies against oral cancer, without any impact on patients’ health [68].

## 9. Application of 3D tumoroids in HNSCC Study

The in vitro pharmacological study on tumoroids reveals the answers used in clinical practice for the treatment and treatment of tumors. This methodology would seem to give a positive response to the study of very aggressive tumors by monitoring the invasiveness of the surrounding tissues in vitro [69]. In some types of cancer such as squamous cell carcinoma of the head and neck ( HNSCC) the aggressive invasion of the surrounding tissues represents the primary cause of morbidity, which is why numerous in vitro studies are underway through the cultivation of tumoroids, in order to provide new therapies that could be complementary to standard anti-proliferative agents [70].

In a study published last May, researchers through direct cultivation of tumoroids resulting from squamous cell carcinoma of the head and neck have validated new discoveries. The results of the research show that triple depletion of the interacting complex CDC37/HSP90α/HSP90β reduces the pro-malignant activity of oral cancer cells. In fact, metastatic cells of oral carcinoma secrete vesicles, exosomes, with a high concentration of HSP90, heat shock proteins. These vesicles are then supplemented by adjacent cells promoting the epithelial-mesenchymal transition (EMT). EMT involves phenotypic changes within the cells going to activate tumorogenesis, promoting the migration and invasion of cancer cells, and activating drug resistance. In the same way EMT changes the polarity of type M2 in the macrophages associated with the tumor. According to the authors of the revolutionary research, triple silencing mediated by CDC37/HSP90α/HSP90β siRNA reduced EMT in oral cancer cells. It follows therefore that the reduction of the epithelial-mesenchymal transition decreases the levels of HSP90 and therefore reduces the malignancy in the receiving cells. Thanks to this it is possible to monitor the invasiveness of the tumor [71].

Instead, in their study, Melissaridou and colleagues used five HNSCC-derived cell lines to study responses to anticancer therapy in 3D and 2D cultures to evaluate cell proliferation, response to anticancer therapy, but also evaluation of the expression of EMT and CSC genes. Based on the protein expression associated with the EMT and the culture time, the spheroids created had variable sizes and densities with characteristics biologically similar to the original tumor. An additional element was also the adjustment up of CDH1, NANOG, and SOX2 in the 3D system rather than in 2D. In this case the pharmacological treatment was carried out with cisplatin and cetuximab, to which the tumoroids showed a reduced sensitivity. Thanks to these results, due to the considerable differences both in terms of gene expression and pharmacological response, it is suggested that the 3D culture model is a completely superior model for studying this type of neoplasm compared to 2D cell cultures [72].

In a further in vitro study on tumoroids deriving from squamous cells of head and neck cancer, the efficacy of the drugs cisplatin, carboplatin, cetuximab, and radiotherapy on the treatment of the tumor itself was assessed. This 3D cellular panel that represented all the genetic and physiological characteristics of the tumor in vivo gave good results, forming scientific proof of the validity of the tested drugs [73].

Head and neck squamous cell carcinomas (HNSCC) are commonly resistant to therapies; therefore the search for predictive markers and new essential elements for a correct treatment is essential. In order to understand how the head and neck cancer cells (HNSCC) responded to specific therapies, Tanaka et al., recreated a 3D predictive tumor model, in other words, creation of tumor organoids from tumors of individual patients. Through this study, they assessed the growth characteristics and above all the response to different pharmacological treatments, with the aim of predicting the response to specific treatments. These organoids showed histological and biological characteristics similar to those of the original tumor as well as the expression of the markers. The tumoroids had been exposed to cisplatin or docetaxel allowing defining of the sensitivity and resistance to the drug, in particular, almost all the organoids showed particular resistance to cisplatin. In addition, these organoids derived from patient tumors have undergone whole genome sequencing and phosphoproteomic analysis in order to identify and study biomarkers useful for patients with HNSCC [71].

The easy access of the biopsy to the original tissue allows the generation of 3D cell cultures from HNSCC cancer cells a quick and successful process. The rapid growth and high efficiency of the HNSCC organoids are thought to derive from the p53 mutation commonly present in this tumor type, in which immune therapy tests can easily be performed.

Various tests have been used on these organoids as a study of chromosomal stability, xenotransplantation, and new pharmacological and conventional tests of chemotherapy/radiation (chemo/RT).

In the study carried out by Hill and D’Andrea, the importance of testing an entire panel of drugs for HNSCC such as cisplatin, docetaxel, and fluorouracil, as well as targeted experimental agents on these organoids, was underlined, in order to understand if a drug is targeted. To get a general and complete picture, pre-treatment and post-treatment patients were selected, in suitable conditions to be able to biopsy the tissue and evaluate the effects and consequences of the induced treatment.

This type of experimentation could establish whether the chemo/RT sensitivity of the organoids actually corresponds to the chemo/RT sensitivity of the patient in the clinic, could allow the identification of molecular mechanisms of tumor resistance. As well as a situation in which the pretreatment tumor organoid is resistant to chemo/RT and the patient has clinical resistance corresponding to the same combination, it could lead to an adequate change in therapy. The study of these post-treatment organoids could provide information to also generate alternative therapies to conventional ones, as well as drug-resistant ones are important because they will accelerate the development of new drugs and combinations of drugs. In this study, the foundations for a future study were established in which the chemo/RT organoid/RT response and the patient’s clinical response can be compared directly, certainly leading to important advances in diagnostics and treatment in HNSCC. [74].

## 10. Current Limitations of Organoids

Organoids have been reported to mimic tissues they are collected from. Some issues have been pointed out about how cells self-organize into the organoids, hypothesizing that these models are unable to guarantee the exact replication of the organ dimensions (i.e., size and shape), and its cellular composition, up to the phenotypic and molecular characteristics [52]. Nevertheless, the current technologies and knowledge are far from producing organoids involving complex and extensive vascular networks supported by vascular smooth muscle cells (VSMCs). Therefore, the main concern is related to a reliable use of organoids in study models of pathologies based on inflammatory conditions; in fact, tissue inflammation needs a proper vascularization to be established and to recall in situ immune cells. Researchers have tried to overcome this issue by means of spinning bioreactors for organoids production: this method is able to promote the vascularization process, especially if MSCs are co-cultured with endothelial cells that can typically generate vascular-like networks.

Another limitation of organoids is the limited presence of well-differentiated ECM compounds: the organoid models require strong and careful ECM-guided growth to have the surrounding tissue similar to the primary organ they are made from. A lacking ECM complexity can lead to a poor immune response, resulting in a concrete limitation for studies involving organoids to assess tumor infiltration and drug penetration [50].

Further studies have reported that organoids from induced pluripotent stem cells (iPSCs) organoids can grow with a limited development in size and morphology. In more detail, MSCs and iPSCs can be differentiated using similar protocols [62], but iPSCs-derived organoids do not reproduce the exact morphology and histology observed in adult tissues: this means that iPSCs-derived organoids may not be able to properly reproduce physiology and pathological characteristics of primary tissues, thus representing an unreliable tissue model. This concept particularly applies to lung cancer, where the use of iPSCs cells, rather than MSCs directly harvested from cancer, would seem to be an issue that makes such tumoroids not useful in cancer research [75,76,77].

Finally, an important limitation is related to the translational applications of organoids in the in vivo therapies. In fact, several applied models to use organoids in human models have involved a scaffold made of extracellular matrix, usually unsuitable for human applications [75]. Besides, culture medium needs to be further improved for long-term expansion, so as to be used for organoid applications in humans [77].

## 11. Conclusions

According to the recent literature, the use of organoids has improved the in vitro research on several aspects of tumor growth and on the therapeutic mechanisms working on molecular and metabolic pathways. In this context, 3D tumoroids have shed light on the cell–cell and cell–matrix interactions that develop during tumor growth and progression; through their culture and manipulation, scientists have understood the role of tumor niche and the reciprocal interactions among cancer cells, stem cells, and the surrounding microenvironment. Molecular and genetic stability of tumoroids has allowed describing the complex interactions among drugs and tumor cells, taking into consideration the role of the extracellular environment and the impact of extracellular vesicles released during cancer and after therapies. Tumoroids have reliably reproduced in vitro the architecture and the main functions of cancers, demonstrating amazing similarities with the characteristics of tumors developed in vivo. Despite all these promising aspects, the in vitro development of tumoroids has been considered somewhat complicated and ethically controversial. In fact, up to now, the literature lacks a standardized and predictable protocol related to the growth and differentiation of different tumoroids. The future efforts should be provided towards a consensus on limits and uses of tumoroids in drug discovery and for targeted therapies: this will be possible, as tumoroids are fully able to preserve both the histological structure and the molecular panel of the human tumors from which they originated.

## Figures and Tables

**Table 1:** Tumoroids: in vitro and in vivo studies.

Study Model	Type of Cancer	Therapeutic Approach	Main Results	References
Engelbreth-Holm-Swarm mouse sarcoma cells	Breast cancer	Chemotherapy	Tumoroids had similar morphology and gene expression of patients with breast cancer	Lee et al. (2007)
Cells of colorectal adenocarcinoma	Metastatic colorectal adenocarcinoma	Chemotherapy	KRAS protein increases cell growth and mitotic activity.	Mousavi et al. (2019)
Human tumoroids were injected into the murine mucosa	Rectal cancer	Chemotherapy	The grafted tumoroids showed equal sensitivity to therapies administered in patients	Ganesh et al. (2019)
Cells of colorectal carcinoma	Colorectal carcinoma	Chemotherapy	3D-tumoroids represent a valid in vitro approach to validate new drug therapies	Finnberg et al. (2017)
3D model called neoplastic brain organoid	Brain tumors	Chemotherapy	NeoCOR showed better results as a 3D model for clinical studies on brain tumors	Shane t al. (2018)
Cancer cells in different 3D tumoroids	Human Primary Liver Cancer	Chemotherapy	SCH772984 inhibited the activation of the ERK protein and demonstrated to play a crucial role in the tumorigenesis	Broutier et al. (2017)
Cultured tumoroids in mice	Human Primary Liver Cancer	Chemotherapy	SCH772984 reduced tumor growth in mice treated with this drug	Broutier et al. (2017)
Cultured tumoroids	Colorectal and liver cancer	Chemotherapy	Organoids maintain the biological characteristics of their original tumor	Jansen et al. (2019)
ADSCs in tumoroid environment	Different cancers	Chemotherapy	The ADSCs maintain their genetic stability and retain all the physiological characteristics of their original tissue.	Huch et al. (2015)
Tumoroids with ESCs and iPSCs in mice models	Pancreatic Tumor	Chemotherapy	ESC and iPSC tumoroids generated functional pancreatic cells	Hohwieler et al. (2019)
Different cell lines in different tumoroids	Different cancers	Chemotherapy	3D tumoroids were able to release tumor-related exosomes	Takanori Eguchi et al. (2018)
Oral Mucosal Organoids	Oral squamous cell carcinoma	Chemotherapy	Oral cancers tumoroids are a smart platform for personalized therapy	Driehuis et al. (2019)
Cell Carcinoma Organoids	Head and Neck Squamous Cell Carcinoma	Chemotherapy	Tumoroids can reproduce both genetic and molecular characteristics of the primary tumors	Hill et al. (2019)
31 lines of tumoroids derived from squamous cell carcinoma of the head and neck (HNSCC)	Squamous cell carcinoma of the head and neck (HNSCC)	Chemotherapy	Tumoroids improve studies on personalized approaches to HNSCC	Driehuis et al. (2019)
Organoids from healthy tissue and tumor tissue	Squamous cell carcinoma of the head and neck (HNSCC)	Photodynamic therapy	Photodynamic therapy can influence epidermal growth factor (EGFR) and tumor growth	Driehuis et al. (2019)
Organoids from tumor tissue	Esophageal carcinoma, Squamous cell carcinoma of the head and neck (HNSCC)	Chemotherapy	Tumoroids were used to test drugs against the onset of HNSCC by suppression of IL-6	Karakasheva et al. (2018)
Metastatic cells of metastatic colorectal and gastro-esophageal carcinoma	Colorectal and gastro-esophageal carcinoma	Chemotherapy	3D tumoroids were used to test sensitivity and specificity to different drugs	Vlachogiannis et al. (2018)
